# Effect of 10 UV Filters on the Brine Shrimp *Artemia salina* and the Marine Microalga *Tetraselmis* sp.

**DOI:** 10.3390/toxics8020029

**Published:** 2020-04-10

**Authors:** Evane Thorel, Fanny Clergeaud, Lucie Jaugeon, Alice M. S. Rodrigues, Julie Lucas, Didier Stien, Philippe Lebaron

**Affiliations:** Sorbonne Université, CNRS, Laboratoire de Biodiversité et Biotechnologie Microbiennes, LBBM, Observatoire Océanologique, 66650 Banyuls-sur-Mer, France; evane.thorel@obs-banyuls.fr (E.T.); fanny.clergeaud@hotmail.fr (F.C.); lucie.jaugeon@sb-roscoff.fr (L.J.); alice.rodrigues@obs-banyuls.fr (A.M.S.R.); lucas@obs-banyuls.fr (J.L.); didier.stien@cnrs.fr (D.S.)

**Keywords:** UV-filters, toxicity tests, marine microalgae, *Artemia salina*, marine environment

## Abstract

The presence of pharmaceutical and personal care product (PPCP) residues in the aquatic environment is an emerging issue due to their uncontrolled release through gray water, and accumulation in the environment that may affect living organisms, ecosystems and public health. The aim of this study is to assess the toxicity of benzophenone-3 (BP-3), bis-ethylhexyloxyphenol methoxyphenyl triazine (BEMT), butyl methoxydibenzoylmethane (BM), methylene bis-benzotriazolyl tetramethylbutylphenol (MBBT), 2-ethylhexyl salicylate (ES), diethylaminohydroxybenzoyl hexyl benzoate (DHHB), diethylhexyl butamido triazone (DBT), ethylhexyl triazone (ET), homosalate (HS) and octocrylene (OC) on marine organisms from two major trophic levels, including autotrophs (*Tetraselmis* sp.) and heterotrophs (*Artemia salina*). In general, results showed that both HS and OC were the most toxic UV filters for our tested species, followed by a significant effect of BM on *Artemia salina* due to BM—but only at high concentrations (1 mg/L). ES, BP3 and DHHB affected the metabolic activity of the microalgae at 100 µg/L. BEMT, DBT, ET, MBBT had no effect on the tested organisms, even at high concentrations (2 mg/L). OC toxicity represents a risk for those species, since concentrations used in this study are 15–90 times greater than those reported in occurrence studies for aquatic environments. For the first time in the literature, we report HS toxicity on a microalgae species at concentrations complementing those found in aquatic environments. These preliminary results could represent a risk in the future if concentrations of OC and HS continue to increase.

## 1. Introduction

In recent decades, sunscreen production has continuously increased with the rise of awareness to protect the skin against damaging sunlight exposure and to reduce the risk of skin cancer [1,2]. Of the many chemical compounds contained in sunscreen products, the active ingredients are ultraviolet (UV) filters, whose purpose is to absorb or reflect UVA and/or UVB radiations ranging from 280 to 400 nm [3].

In 2016, 60 different UV filters were reported on the market; these compounds are subject to different regulations around the world [4]. UV filters are regularly detected in various aquatic environmental compartments, including lakes, rivers, surface marine waters and sediments [3,5,6,7]. Some UV filters have been investigated more than others in occurrence studies, such as benzophenone-3 (BP3), ethylhexyl methoxycinnamate (EMC), octocrylene (OC) and 4-methylbenzylidene camphor (4-MBC). In marine coastal waters, most of the filters occur at concentrations in the range of 0.1–10 µg/L [5,6,7]. Concentrations as high as 1.4 mg/L was reported for BP3 in the U.S. Virgin Islands’ coastal waters [8]. These chemicals can enter in the marine environment in two ways: either indirectly from the effluent of wastewater treatment plants, or directly from swimming or recreational activities [9]. Furthermore, their lipophilic nature results in low water solubility, high stability and tendency to bioaccumulate [10,11].

To investigate the impact of these UV filters on the environment, ecotoxicological studies have examined various trophic levels, from microalgae, coral to fish. Several studies have demonstrated that some of these compounds can disrupt survival [12,13,14]. behavior [15,16], growth [14,17,18], development [19,20], metabolism [21,22,23], gene expression [24,25] and reproduction [15,26,27] in various species. It should be noted that most toxicological studies on organic UV filters were conducted on BP3, EMC and 4-MBC [7]. BP3 and EMC have been banned in Hawaii and Key West, Florida The Republic of Palau and U.S Virgin Islands have also banned these two UV-filters, as well as OC. The adoption and implementation of European legislation on the registration, evaluation, authorization and restriction of chemicals (REACH) requires several additional ecotoxicity data and promoting the use of invertebrates as models for toxicity assays [28].

The aim of this present study was to evaluate the toxicity of ten common UV filters: benzophenone-3 (BP-3), bis-ethylhexyloxyphenol methoxyphenyl triazine (BEMT) butyl methoxydibenzoylmethane (BM), diethylaminohydroxybenzoyl hexylbenzoate (DHHB), diethylhexyl butamido triazone (DBT), ethylhexyl salicylate (ES), ethylhexyl triazone (ET), homosalate (HS), methylene bis-benzotriazolyl tetramethylbutylphenol (MBBT) and octocrylene (OC) on two model organisms commonly used in ecotoxicity assays [22,29,30,31]: the green algae *Tetraselmis* sp., a primary producer commonly used for chronic algal toxicity [22] and the brine shrimps *Artemia* spp. (here *A. salina*) readily available worldwide and easy to breed. Any alterations in these populations may result in chain reaction effects on organisms at higher trophic levels [22,31]. This study is a preliminary study before investigating a large diversity of species.

## 2. Materials and methods

### 2.1. Test Substances and Experimental Solutions

The UV filters benzophenone-3 (BP-3), bis-ethylhexyloxyphenol methoxyphenyl triazine (BEMT), butyl methoxydibenzoylmethane (BM) and methylene bis-benzotriazolyl tetramethylbutylphenol (MBBT) were purchased from Sigma–Aldrich (Saint-Quentin Fallavier, France). 2-Ethylhexyl salicylate (ES), diethylaminohydroxybenzoyl hexyl benzoate (DHHB), diethylhexyl butamido triazone (DBT), ethylhexyl triazone (ET), homosalate (HS) and octocrylene (OC) were provided by Pierre Fabre Laboratories (Touluse, France).

Before each toxicity test—and due to the low water solubility of the compounds—stock solutions at 1 mg/mL were prepared by dissolving each UV filters in dimethyl sulfoxide (DMSO, Sigma–Aldrich, purity >99%). These solutions were diluted in order to add the same amount of DMSO to all samples and to obtain exposure concentrations ranging from 20 ng/L to 2 mg/L for *A. salina*, and 10 µg/L to 1 mg/L for *Tetraselmis* sp. The lower concentrations tested were roughly those reported in marine ecosystems (0.1–10 µg/L). For *Tetraselmis* sp., three concentrations were selected to investigate a possible dose-response effect. The lowest-tested concentration was the highest concentration reported in the environment for most filters. This assay on *Tetraselmis* sp. is considered as a preliminary study before investigating the response of phytoplankton on a wide phylogenetic diversity of species. DMSO concentration in the experiments was always 2.5% (v/v). A DMSO control (2.5% v/v) and a blank control were also included. The blank control was artificial seawater (ASW) for *A. salina*, and growth medium for *Tetraselmis* sp.

### 2.2. Artemia salina Mortality Test

*A. salina* cysts were purchased from AquarHéak Aquaculture (Ars-en-Ré, France) and stored at 4 °C. Dried cysts were hatched in a constantly aerated transparent V-shaped hatching incubator filled with 500 mL of artificial seawater (ASW) at a salinity of 37 g/L, prepared with Instant Ocean salt (Aquarium Systems, Sarrebourg, France). Incubation was carried out for 48 h, at 25 °C under continuous light the first 24 h. A 12:12 h light regime was then applied until the nauplii reached the instar II–III stage. Ten nauplii were transferred into 5 mL glass tubes filled with ASW (2 mL) supplemented with DMSO and UV filters. The tubes were incubated at 25 °C under a 12:12 h light regime. The experiments were performed in sextuplicate. During the exposure period, there was no aeration and the nauplii were not fed. The mortality rate was estimated after 48 h by counting the dead nauplii under stereoscope. Organisms with no swimming activity or movement of appendices for 10 s even after mechanical stimulation with a Pasteur pipette were counted as dead. The tests were considered valid if the control’s average mortality rate was < 20%.

### 2.3. Tetraselmis sp. Toxicity Test

#### 2.3.1. Experimental Procedure

*Tetraselmis* sp. (RCC500) was purchased from the Roscoff culture collection and was grown in filtered (pore size: 0.22 µm) and autoclaved seawater enriched with a 50-fold diluted f/2 medium (Sigma–Aldrich). The culture was maintained under controlled conditions at 18 °C (±1 °C) with a photon flux of 70 µmol photons. m^−2^.s^−1^ under a dark:light cycle of 12:12 h. Toxicity tests were conducted in 150 mL Erlenmeyer flasks containing 50 mL of culture. Algae cells in exponential growth phase were used as inoculum with an initial cell density of 5.10^4^ cells/mL. Three replicates per UV filter concentration were performed. After 7 days of exposure, different morphologic and physiological cells properties were monitored via flow cytometry (FCM). Analyzed parameters were granularity, relative cell volume, chlorophyll a fluorescence, esterase activity and growth. The control experiment was a *Tetraselmis* sp. culture supplemented with DMSO (2.5%).

#### 2.3.2. Flow Cytometry (FCM) Analyses

Aliquots were collected after seven days of exposure to be analyzed in a FACSCanto II flow cytometer (Becton Dickinson, Franklin Lakes, NJ, USA) equipped with an air-cooled argon laser (488 nm, 15 mW). To characterize the microalgae population—and to exclude non-algal particles—the forward scatter (FSC, an estimation of cell size) and side scatter (SSC, an estimation of granularity) dot-plots were established before each measurement. The flow rate of the cytometer was set to low with an acquisition time of 1 min.

The data recorded by FCM were measured directly (autofluorescence, granularity, size) and indirectly by the use of fluorochromes (esterase activity). Cellular density was determined using Becton Dickinson Trucount^TM^ (San Jose, CA, USA) 10 μm beads for calibration, as already described [32]. Growth rate (µ), expressed as day^−1^ were calculated using the following equation: µ = (ln(N_t_)–ln(N_0_))/(t–t_0_), where N_t_ is the cell density at time t and N_0_ is initial cell density. Chlorophyll a autofluorescence was measured and detected in the FL3 channel. Relative cell volume (size) and granularity were directly estimated with the forward light scatter (FSC channel) and with the side scatter channel (SSC), respectively. The metabolic activity was determined based on the esterase activity. Cellular esterase activity measurement is a method frequently used to determine the metabolic activity of cells [33]. The most common substrates used are acetomethyl esters (calcein-AM and (BCECF-AM) and fluorescein diacetate (FDA) and its various substituted derivatives. Once inside the cell, the substrate is converted by intracellular esterases into calcein or fluorescein that has a net negative charge at neutral pH. Thus, the fluorescent molecules are maintained inside the cell by the intact membrane potential and therefore, the concentration of fluorescein (or derivatives) trapped in the cell increases with time. In this study cells were stained with the fluorochrome Chemchrom V6 (10-fold diluted in ChemSol B26 buffer—Biomérieux, Marcy l’Etoile, France) at 1% final concentration, and incubated for 15 min at room temperature in the dark before analysis using the FL1 channel. All cytometry data were analyzed using BD FACSDiva (Becton Dickinson, San Jose, CA, USA). Results were expressed as percentage of variation relative to the control (100%).

### 2.4. Statistical Analysis

Results are reported as mean and standard deviation (SD), calculated from the 3 or 6 replicates. For both tests and all the parameters measured, differences between controls and nominal concentrations of UV filter were analyzed using R software, by one-way analysis of variance (ANOVA) followed by post-hoc Tukey HSD tests for pairwise comparisons. In all cases, significance was accepted when *p* < 0.05. Half maximal lethal concentration (LC_50_) is the concentration that induces 50% mortality in *A. salina* (for BM, HS and OC) or *Tetraselmis* sp. (for HS only). Half maximal effective concentration (EC_50_) refers to the concentration that induces a response halfway between the baseline and maximum. EC_10_ corresponds to the concentration that affect 10% of the population. LC_50_/EC_50_-values were estimated with a log(agonist) vs. response—Variable slope (four parameters) regression model in Prism 5 (GraphPad Software Inc., San Diego, CA, USA).

## 3. Results and Discussion

### 3.1. Effects on Artemia salina Mortality

**Figure 1:**
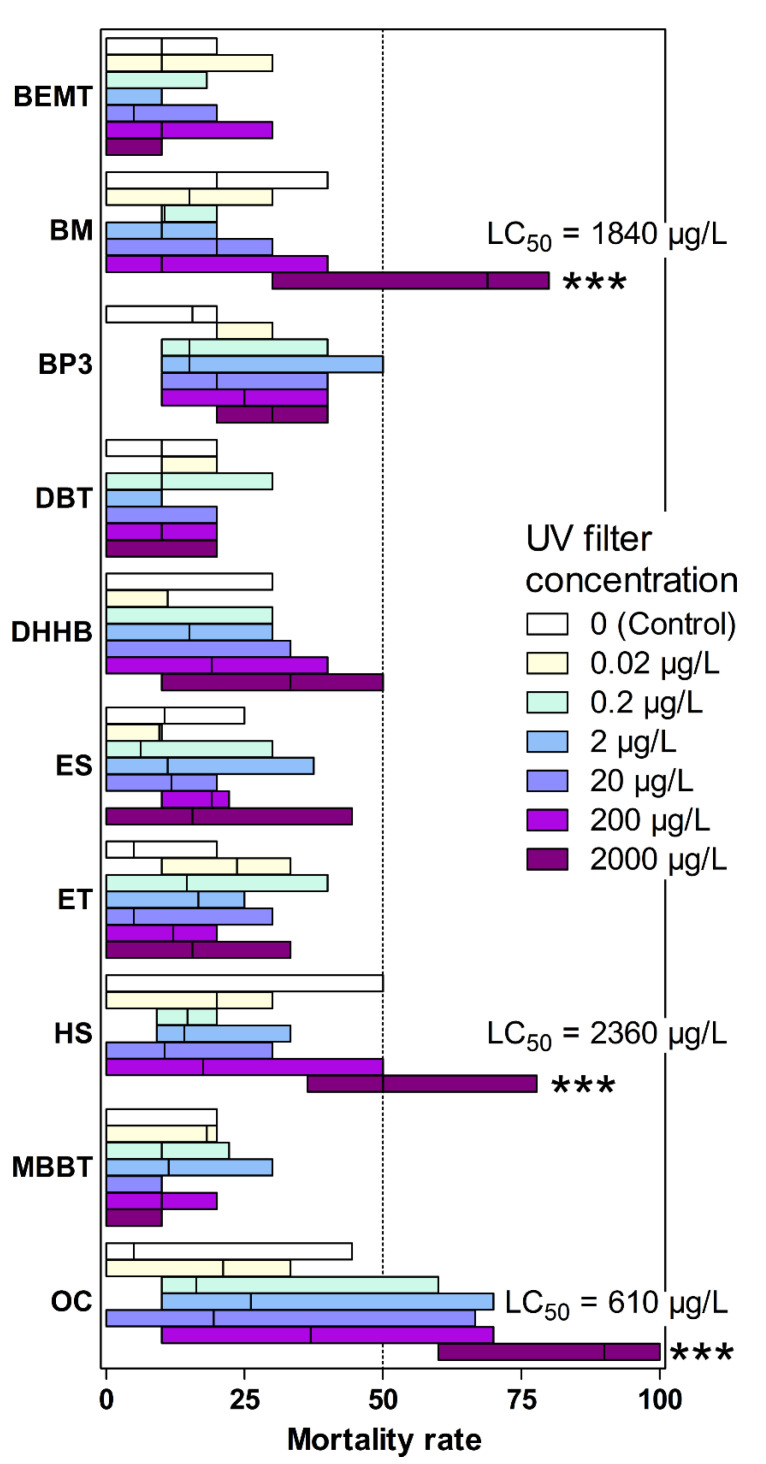
Mortality rate of *A. salina* exposed to the 10 UV filters at 6 concentrations. Boxes delineate the minimal and maximal values and the vertical line is the median of six replicates. Significance levels relative to control determined by ANOVA followed by the Tukey’s multiple comparison test: *** *p* < 0.001. Results were not significant unless otherwise stated. For BM, HS and OC, the LC_50_ is reported on the figure.

The toxicity of several organic UV filters on the marine crustacean *Artemia salina* (Nauplii Instar II/III) was determined after a 48-h exposure by counting dead larvae (Figure 1). At the highest concentration tested (2 mg/L), HS, BM and OC demonstrated a significant effect on Nauplii survival (*p* < 0.05) with mortality values reaching 54 ± 16%, 64 ± 19% and 88 ± 16%, respectively. At lower concentrations of these filters no significant effect was detected. For BP3, BEMT, MBBT, ES, DHHB, DBT and ET no toxicity was observed, even at the highest concentration.

Our results indicate that among the different UV filters tested in this study, OC was the most toxic molecule showing the lowest LC_50_ concentration (0.6 mg/L), followed by BM and HS (1.8 mg/L and 2.4 mg/L, respectively). Environmental HS and BM concentrations reported so far are at least 500 times lower than LC_50_, with values lower than 3 µg/L [3,5,34,35]. OC concentrations in coastal waters are higher and have been detected up to 9 µg/L [5,35,36]. This is the first report showing OC toxicity on *Artemia salina*. We also observed a concentration-dependent increase in mortality of *Artemia* with respect to the control. This is congruent with the toxicity observed, at lower concentration, on coral (50 µg/L) [23], urchin, mussel and algae (40–80 µg/L) [19]. OC also affects the developmental process in zebrafish [37]. Here the LC_50_ on *A. salina* is approximately 90 times higher than the highest OC concentrations in marine waters reported so far. It should be mentioned as well that OC concentrations in the 50–100 µg/kg range have been frequently reported in sediments [38,39]. OC is a pseudo-persistent pollutant; its contamination of the environment is refreshed daily. As such, it may indeed affect benthic crustacean.

### 3.2. Effects on Tetraselmis sp.

#### 3.2.1. Growth Rate and EC_50_ Values

**Figure 2:**
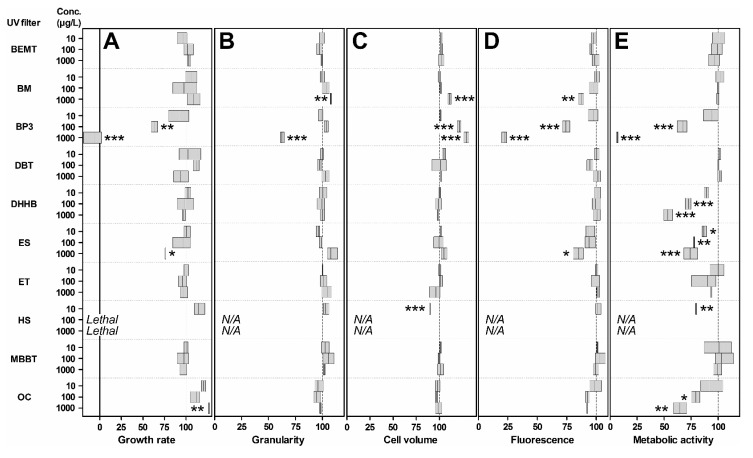
Relative (**A**) growth rate, (**B**) granularity, (**C**) cell volume, (**D**) fluorescence and (**E**) metabolic activity of exposed *Tetraselmis* compared to control, set to 100%. The boxes delineate the minimal and maximal values. The vertical line in the boxes is at mean. Significance levels relative to negative control determined by ANOVA followed by the Tukey’s multiple comparison test: *** *p* < 0.001, ** *p* < 0.01, * *p* < 0.05. Results were not significant unless otherwise stated. N/A: not applicable, the data could not be obtained due to extensive cell death.

After 7 days of exposure, HS, BP3 and ES induced a significant decrease of algae growth (Figure 2A). The growth rate of algae exposed to ES at 1 mg/L decreased by 24% compared to control (*p* < 0.05). Data for the presence of ES in the environment are scarce. ES concentrations in coastal seawater have been reported in the range 1–1030 ng/L [5,40]. At these concentrations our results suggest that ES may have no impact on *Tetraselmis* sp.; it is crucial though to determine its toxicity on a wide range of phytoplankton species before concluding. For BP-3 we observed a concentration dependent decrease in growth, which was statistically significant at 100 µg/L (*p* < 0.01) and 1 mg/L (*p* < 0.001). At 1 mg/L, the growth rate was negative, which translates in a decreased cell concentration compared to t_0_. The 7-day EC_50_ value for BP3 was 143 µg/L. BP3 EC_50_ values on several microalgae species have been reported previously to be roughly in the 100 µg/L to 20 mg/L range [17,21,41,42]. Seawater BP3 concentrations in the µg/L range have been frequently reported in the literature [5,10]. Extremely high values of 1.4 and 0.6 mg/L have been recorded in the U.S. Virgin Islands [8]. At such high concentrations, BP3 is highly toxic for *Tetraselmis* sp. Finally, the most important decline was with the UV filter HS. No algal cells were detected in the presence of HS at 100 µg/L and 1 mg/L. LC_50_ with HS was estimated at 74 µg/L, while it has been shown that HS concentration in aquatic environments can reach ~3 µg/L [5,35]. Further experiments should be conducted on a wider diversity of phytoplankton species and at lower concentrations to better interpret the toxicity of HS and its potential impact of phytoplankton communities. If toxic to Symbiodiniaceae, HS may also contribute to coral bleaching. OC induced a slight but significant increase for the growth rate at 1 mg/L. The increased growth rate may be due to a hormesis effect. Nonetheless, the decreased metabolic activity at day 7 is a significant sign of toxicity detectable at 100 µg/L and above. Similar differences in the response of different physiological parameters were already reported [21] for the toxicity of BP3 in the microalgae *Chlamydomonas reinhardtii*. The growth rate of *Tetraselmis* was not affected by BEMT, BM, DBT, DHHB, ET and MBBT, even at 1 mg/L. To the best of our knowledge, there are no data on the toxicity of these filters on phytoplankton species. As mentioned above, these results should be considered as preliminary data and more assays should be performed on a wide diversity of species. Co-occurrence of these filters should be investigated with many sunscreen brands containing different UV filters and often a few UV filters in the one product.

#### 3.2.2. Impact on Cell Morphology

Three of the UV filters induced cell morphologic alterations (Figure 2B,C). Cells cultured in the presence of BM have experienced a significant increase in cell volume and granularity at 1 mg/L (*p* < 0.05). This concentration is 1000 to 10,000 times higher than the few concentrations reported in the field [3,5,34]. According to environmental concentrations of other UV filters, one can assume that the effective concentration of 1 mg/L is probably higher than any BM environmental concentration, but this remains to be confirmed. BP3 caused a dose dependent increase of relative cell volume at 100 µg/L and above, reaching up to 129% of control cell volume at 1 mg/L (*p* < 0.001). Meanwhile, this UV filter induced a significant 38% granularity decrease at 1 mg/L (*p* < 0.001). With reference to the environmental concentrations of BP3 (see above), this filter should exert a significant impact on phytoplankton communities. These results are congruent with what was recently reported [42] on *Arthrospira* sp. With HS, cell volume and granularity could not be measured at 100 and 1000 µg/L. However, a significant cell volume decrease was observed at 10 µg/L of HS (−10.2%, *p* < 0.001). Here, the EC_10_ value is slightly lower than 10 µg/L, i.e., within the same order of magnitude than the highest water column concentration reported so far [5]. Again, it is expected that HS should affect microalgae communities in bathing areas. No significant effect was observed for BEMT, DBT, DHHB, ES, ET, MBBT and OC. As mentioned above, there are no data on the toxicity of these filters on the morphology of phytoplanktonic cells but further investigations on other species are needed to conclude on their toxicity on phytoplankton.

#### 3.2.3. Impact on Autofluorescence

The results of FCM analysis revealed that several UV filters significantly reduced chlorophyll a (Chl a) cell fluorescence (Figure 2D). The decrease was significant with BM (–13%, *p* < 0.01) and ES (–15%, *p* < 0.05) at 1 mg/L. A strong dose-dependent autofluorescence inhibition was observed upon exposure to BP3 at concentrations of 100 µg/L (*p* < 0.001) and above. Inhibition reached 78% at the highest dose. Again, autofluorescence could not be measured in cells treated with HS at 100 and 1000 µg/L due to the cell degradation at these concentrations. No significant effect was observed for BEMT, DBT, DHHB, ET, MBBT and OC. Again, this is the first report that shows results on the effect of BEMT, MBBT, DHHB, ET and DBT on phytoplankton. These results further demonstrate that it is important to follow different parameters since OC for instance has no impact on the fluorescence but has an impact on the growth rate and metabolic activity.

#### 3.2.4. Impact on Cell Metabolic Activity

Metabolic activity was determined by estimating the relative esterase activity in exposed cells compared to control. It was measured by CV6 staining and highlighted significant decreases in metabolic activities with half of the tested UV filters (Figure 2E). Algae exposed to ES and HS experienced a decreased esterase activity at 10 µg/L. The effect of BP3, DHHB and OC was significant at 100 µg/L and above. A similar decreased esterase activity was reported for *C. reinhardtii* exposed to BP3, although at concentrations in the mg/L range [21,43]. For DHHB, the effect was only observed for the microalgae and for the esterase activity but not for other parameters. Therefore, the environmental risk cannot be estimated since natural concentrations have never been reported for this UV filter. No significant effect was observed for BEMT, MBBT, DBT, ET and BM.

The toxicity of the different UV filters is not the same between phytoplankton and zooplankton and probably varies between species within the same group. This means that it is important to study the response of several species within a given group. For *Tetraselmis* sp., we see that the toxicity vary according to the examined parameters. Therefore, it is important to measure several parameters. For example, this is clear when we look at the results of the fluorescence parameter. This could imply that when the impact of UV filters on corals is analyzed based on the simple fluorescence parameter of the zooxanthellae (symbionts), this approach is too simplistic because zooxanthellae can perhaps react on other parameters than fluorescence.

## 4. Conclusion

The present work demonstrates that several UV filters exert toxicity on *A. salina* and *Tetraselmis* sp. HS was the most toxic UV filter for the microalgae. EC_50_ was 74 µg/L and significant adverse effects were recorded at the lowest concentration tested (10 µg/L). HS was also toxic for *A. salina*, although at much higher concentrations (LC_50_ 2.4 mg/L). Since HS concentrations up to three µg/L have been reported in aquatic environment, HS may represent a potential risk for marine phytoplankton communities. Further research is needed to investigate the HS toxicity with a larger diversity of phytoplankton species.

OC was toxic on both models with a dose-dependent effect on the microalgae. OC significantly altered *Tetraselmis* sp. metabolic activity at 100 µg/L. On *A. salina*, LC_50_ was 610 µg/L. Overall, OC toxicity was observed at 100 µg/L with these models. The toxicity of OC occurred at concentrations 90 (*A. salina*) and 15 (*Tetraselmis* sp.) times higher than the highest environmental concentrations reported so far. These results highlight a potential toxicity of OC on marine organisms as input concentrations continue to increase. BM was toxic towards the brine shrimp at high concentrations with a LC_50_ of 1.84 mg/L and had little effect on the microalgae. BM toxicity was observed at one mg/L for the algae. Such high concentrations has never been reported in the occurrence studies, although the presence of this filter should be monitored in a large range of ecosystems to better interpret its potential toxicity. ES, BP3 and DHHB had a significant impact on the microalgae metabolic activity at concentrations between 10 and 100 µg/L but had little effect on *A. salina*

Overall, this research supports the need of establishing environmental quality standards for UV-filters based on toxicity testing with key marine organisms, as well as identifying and reducing potential toxic UV filters from entering the environment. There are still many UV filters for which environmental concentrations are missing therefore the potential to estimate the environmental risks are still lacking for coastal ecosystems. Based on toxicity results, it is urgent to design new environment friendly UV filters.
